# Heat Stress Responses in Birds: A Review of the Neural Components

**DOI:** 10.3390/biology10111095

**Published:** 2021-10-25

**Authors:** Mark W. Bohler, Vishwajit S. Chowdhury, Mark A. Cline, Elizabeth R. Gilbert

**Affiliations:** 1 Department of Animal and Poultry Sciences, 2160 Litton-Reaves Hall, Virginia Polytechnic Institute and State University, Blacksburg, VA 24061, USA; Mwbohler@vt.edu (M.W.B.); macline2@vt.edu (M.A.C.); 2 Laboratory of Stress Physiology and Metabolism, Faculty of Arts and Science, Kyushu University, Fukuoka 819-0395, Japan; vc-sur@artsci.kyushu-u.ac.jp

**Keywords:** birds, heat stress, hypothalamus, corticotropin-releasing factor, thyroid hormones, stress, food intake

## Abstract

**Simple Summary:**

Heat stress is a major environmental condition negatively impacting the wellbeing of various avian species. In chickens, heat exposure is associated with disruption of metabolic and immune system function, and an increased risk of mortality. This has a negative impact on the food economy, as chicken products make up roughly 34% of the world’s protein. Techniques to mitigate exposure to high temperatures have been discussed in depth, and most research suggests that the root cause of heat stress-induced physiological aberrations is alterations in the stress response and reduced food intake. Unfortunately, little is known about thermoregulation in birds. That thermoregulation, food intake, and the stress response are all mediated by the hypothalamus make it tempting to speculate that it is the central hub at which these systems interact and signals from diverse pathways are integrated. Thus, this review discusses the neural circuitry in birds associated with thermoregulation, food intake, and stress response at the level of the hypothalamus, with a focus on how these systems might interact in the presence of heat exposure.

**Abstract:**

Heat stress is one of the major environmental conditions causing significant losses in the poultry industry and having negative impacts on the world’s food economy. Heat exposure causes several physiological impairments in birds, including oxidative stress, weight loss, immunosuppression, and dysregulated metabolism. Collectively, these lead not only to decreased production in the meat industry, but also decreases in the number of eggs laid by 20%, and overall loss due to mortality during housing and transit. Mitigation techniques have been discussed in depth, and include changes in air flow and dietary composition, improved building insulation, use of air cooling in livestock buildings (fogging systems, evaporation panels), and genetic alterations. Most commonly observed during heat exposure are reduced food intake and an increase in the stress response. However, very little has been explored regarding heat exposure, food intake and stress, and how the neural circuitry responsible for sensing temperatures mediate these responses. That thermoregulation, food intake, and the stress response are primarily mediated by the hypothalamus make it reasonable to assume that it is the central hub at which these systems interact and coordinately regulate downstream changes in metabolism. Thus, this review discusses the neural circuitry in birds associated with thermoregulation, food intake, and stress response at the level of the hypothalamus, with a focus on how these systems might interact in the presence of heat exposure.

## 1. Introduction

Heat exposure and heat stress are the most common negative environmental conditions affecting the poultry industry. According to Engormix, heat stress is defined as a form of hyperthermia in which the physiological systems of the body fail to regulate body temperature within a normal range. Heat stress can be caused by a variety of factors including natural (solar exposure, cloud cover, wind, humidity) and artificial (housing temperature, humidity, and air flow) ambient temperatures. When housing conditions are ideal, the environment outside of the house can still influence the internal temperature or humidity, potentially leading to heat stress.

In poultry production, heat stress is responsible for roughly $165 million in losses a year in the U.S. [1] and this number is likely to increase with the current global warming crisis. This not only raises concerns for the industry, but has major implications in the world’s food economy as chicken meat accounts for 34% of the world’s protein production [2]. In egg production, heat stress reduces the numbers of eggs that laying hens produce by 20 [3,4] to 28% [5]. Heat stress is highly associated with increased mortality in housing environments and during transit [6,7,8], and in birds that survive there is usually a reduction in body weight [9,10,11], some level of immunosuppression [11], and impaired metabolism [12,13,14].

There is an overall reduction in the number of eggs laid, egg weight, and shell thickness when laying hens are exposed to heat stress [15], and in hens there is a decrease in plasma calcium concentrations as a consequence of reduced food intake [16]. Despite reduced egg production, the eggs laid tend to be of higher quality [15]. Fertility and hatchability were increased in eggs from laying hens that were inseminated early (1000 h) [17] or late (1800 h) [18] in the day rather than in the middle of the day (1500 h) [17]. It is worth noting that while endogenous factors played a role in these differences, the variation in ambient temperature throughout the day had an effect on reproductive capacity [19].

Broiler-type chickens have undergone intense artificial selection for rapid growth and feed efficiency. This growth is primarily achieved by rapid accretion of muscle protein, particularly in the breast [20]. As a consequence of rapid growth and development, the broilers eat incessantly, necessitating some level of feed restriction at all stages of growth, and this drive to eat is likely associated with a dampened satiety mechanism in that they produce less hunger-suppressing factors such as glucagon-like peptide 1 (GLP-1) and α-melanocyte stimulating hormone (α-MSH) than layer-type chickens [21]. Lastly, broilers have been selected to thrive in temperate environments [22] and are endothermic and precocial at hatch, like other poultry species (For more information we direct you to: [23]). While optimized for production purposes, these selected traits have predisposed the broiler to be more susceptible to heat stress than layers. Animals with body compositions of primarily protein produce more heat, and disperse less than those that develop more fat [24] suggesting that rapid and abundant development of muscle tissue hinders heat dissipation during high temperature exposure. Additionally, the caloric intake required to fuel such rapid growth is associated with excessive heat production due to metabolism and may exacerbate the effects of heat stress.

Many of the physiological impacts of heat stress have been discussed in depth: performance [1,25,26,27], immunosuppression [1,28], impaired metabolism [29], the effect on the industry [30,31], and mitigation techniques [1,28,32,33]. In the reviews referenced above, two common themes associated with heat stress are food intake and the corticotropin-releasing factor (CRF) signaling system. Despite repeated discussion in the heat exposure literature, there has yet to be a synthesis of the information on these systems in the context of high environmental temperature (HAT) exposure. Thus, the purpose of this review is to explore the central components mediating the stress response and food intake in birds exposed to HAT and propose a plausible mechanism through which heat exposure mediates these pathways.

## 2. Development of the Avian Thermostat

### 2.1. Development of Temperature Sensing Neurons

The avian thermostat has been discussed, but primarily in the context of birds exposed to a cold environment [23]. Most research regarding temperature sensing neurons in birds has been conducted in Muscovy ducks [34,35], chickens [36], and turkeys [37], due to their independence at hatch (precocial) and endothermic development just prior to hatch [38]. The remainder of research on thermoregulation has been performed almost exclusively in mammalian model systems. Temperature-sensing neurons are generally considered to be nerves in the periphery that express thermo transient receptor potentials (thermoTRP). ThermoTRPs are a family of ion channels that respond to temperature, however the mechanism through which they open in response to temperature is not well understood. Upon their discovery, it was hypothesized that they opened due to detection of change in membrane voltage following a change in temperature, however the structural component of that sensor was unknown [39]. More recent reports of other temperature-sensitive ion channels report that this voltage-sensing structure is still unknown [40]. Despite the lack of knowledge regarding how these channels open, once activated they are permeable to calcium and sodium ions, and ion influx leads to depolarization [41]. Based on the function of thermoTRPs, these channels open to modulate the activity of neurons and thereby control their temperature sensitivity. To our knowledge, there are no neurons with the primary function of firing due to the presence of high or low temperatures in the brain. The localization of these neurons and their functions have been a source of controversy for many years [42], but some have suggested that thermoTRPs are also expressed in the hypothalamus and respond to the temperature of the surrounding brain tissue and potentially the temperature of blood in nearby blood vessels [43] (also see below, Section 2.2).

The region responsible for detecting changes in temperature and initiating an appropriate response is the preoptic area (POA) of the hypothalamus. Results from studies using Muscovy ducks revealed that temperature-sensing neurons began to come into existence during the last week of the embryonic period and completed their differentiation and maturation by day 10 post-hatch [34,35]. From embryonic day 28 to day 5 post-hatch, the POA was comprised primarily of cold-sensing neurons [34,35]. From day 5 post-hatch to day 10 post-hatch, the neurons began to differentiate into more warm-sensing neurons [34,35]. It is unclear why or how temperature-sensitive neurons shift from cold to warm sensitivity. It is possible that the shift in thermo-sensitivity is linked to a similar phenomenon observed with gamma aminobutyric acid (GABA) receptors. During development, GABA receptors shift from being excitatory in nature to inhibitory, and this occurs due to a change in intracellular chloride concentrations [44]. This shift in the concentration of intracellular ions is a potential means for transitioning towards warm sensitivity, however this hypothesis requires further exploration. Interestingly, there is a third subset of neurons that is found in greater abundance than the warm- and cold-sensing neurons combined, termed temperature guardian neurons [34]. Temperature guardian neurons are not responsive during mild temperature changes but instead are active when temperatures approach near-extreme ends of the spectrum and are thought to be responsible for initiating more robust thermoregulatory mechanisms.

Chickens express a thermoTRP, transient receptor potential ankyrin 1 (TRPA1), in skin which is responsive to temperatures slightly below the average body temperature (41–42 °C) [45]. This ion channel may thus be responsible for detecting exposure to heat and transducing the signal that initiates the heat stress response. Additionally, transient receptor potential vanilloid 1 (TRPV1) is expressed in the majority of heat-sensitive neurons in the dorsal root ganglia, and responds to temperatures 45 °C and above [45], which are borderline lethal for most birds [46]. Unfortunately, there is very little information regarding thermosensation in birds, and thus it is unclear whether TRPA1 or TRPV1 relay information to the same region as in mammals (for more see: [47]). Although similar to mammals in the display of thermoregulatory behaviors, such as panting and wing (limbs in mammals) spreading, the neural circuitry mediating these behaviors are unknown in birds. Additionally, little is known about the neural circuitry regulating drinking behaviors in avian species, although it is likely similar to mammals (see below).

### 2.2. A Brief Mention: The Mammalian Thermosensory Pathway

**Figure 1:**
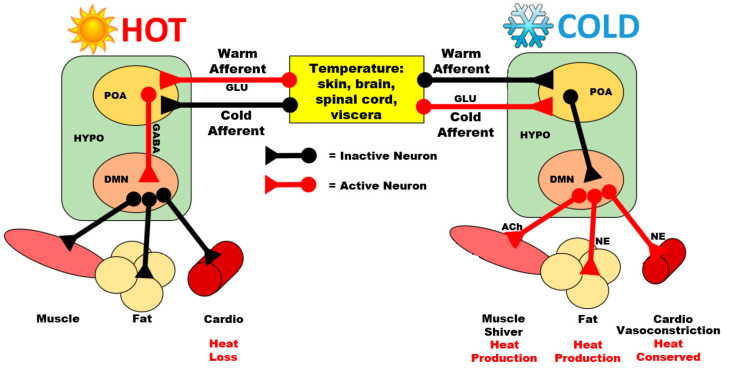
Simplified representation of the mammalian thermoregulatory system. Temperature receptors in various regions relay information to the preoptic area (POA) of the hypothalamus through the release of glutamate (GLU) from warm or cold afferents. For high temperature, the POA inhibits the dorsomedial nucleus of the hypothalamus (DMN) through gamma-aminobutyric acid (GABA) release. This results in the inactivation of circuits to the muscle, fat, and cardiovascular system, thereby reducing heat producing activities and increasing heat loss. For cooler temperatures, POA inhibition of the DMN decreases, which causes increased outflow from the DMN to peripheral targets, resulting in shivering through skeletal muscles, mediated by acetylcholine (ACh) release, increased heat production from fat metabolism due to increased norepinephrine (NE) release, and vasoconstriction to conserve heat. Adapted from [56].

In mammals, the neural pathways are better understood (Figure 1) and can provide insights on birds, in which some pathways are likely conserved. From the thermoreceptors in the periphery, information travels to and up the spinal cord to the parabrachial nucleus [48,49]. The parabrachial nucleus has glutamatergic neuronal projections directly targeting divisions of the POA responsible for sensing cutaneous temperatures and then mediating a response, most commonly initiating vasodilation following heat exposure [48]. Mechanisms mediating more complex heat-loss mechanisms, such as panting, are less understood. These data are further supported by another study [50], which suggests that thermoregulatory behaviors are not mediated by the thalamus [50]. Similar pathways are observed in the control of drinking behavior in mammals. Several signals, including blood pressure, blood volume, and osmolarity, elicit signaling cascades in specific regions of the brain, including the parabrachial nucleus and the POA, which then signal thirst and initiate dipsogenic behavior (for more, see: [51]). Hormonal control of thirst and drinking behaviors are primarily mediated by circulating angiotensin II [52], which is upregulated during heat stress [53]. Angiotensin II regulates thirst at the subfornical organ (SFO) [54] and it was hypothesized that thirst signaling occurs through SFO dopaminergic efferents, some of which terminate in the POA [55].

**Figure 2:**
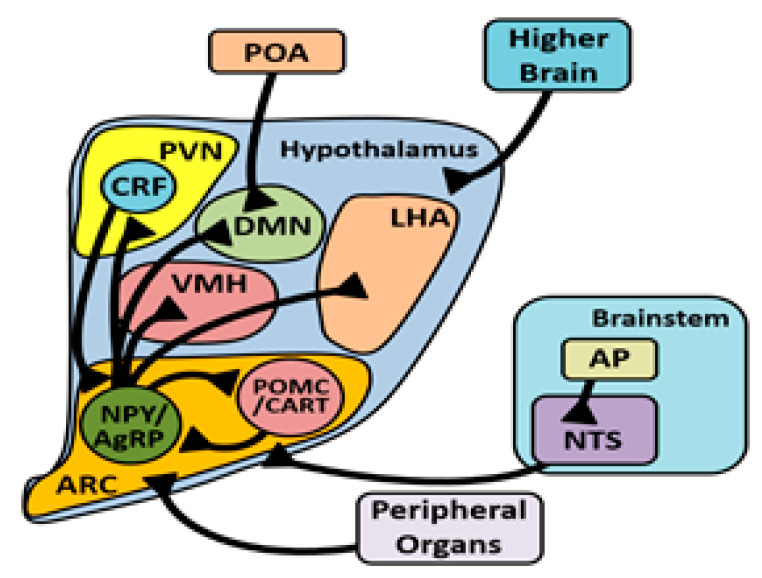
Based on mammalian models, there are projections of NPY neurons from the ARC to other appetite-associated hypothalamic nuclei, reciprocal innervation between NPY and POMC neurons in the ARC, and reciprocal innervation between NPY and CRF in the ARC and PVN, respectively. Peripheral signals influence feed intake by affecting the ARC. Several other inputs from higher brain centers and the brainstem also converge on the hypothalamus and affect appetite. Additionally, temperature-related information from the POA converges on the hypothalamus, although how this connection affects feed intake is unclear. Adapted from [64,65,66].

Thermoreceptors are also found in the brain, primarily the hypothalamus, and are known to elicit similar responses as signals that originated from cutaneous thermoreceptors. TRPV1 is found on cells in the paraventricular nucleus (PVN) and dorsomedial nucleus (DMN) of the hypothalamus of mice [57], as well as in the anterior (AH) and lateral hypothalamus (LH) [58], and in the PVN, DMN, LH, and arcuate (ARC) nucleus of rats [59]. In the 1940s, it was hypothesized that the hypothalamus used food intake to mediate body temperature [60]. That thermoTRPs such as TRPV1 are found on cells located in key hypothalamic nuclei that mediate food intake (Figure 2) suggests that the hypothalamus does not use food intake regulation to mediate body temperature, but rather it responds to thermal stimuli by increasing or decreasing food intake following the opening or closing of thermoTRPs. However, there has been some controversy over the precision of TRPV1 detection and quantification in the rodent brain [57]. Regardless of discrepancies in localization, results from functional studies in the ARC [61] and supraoptic nucleus (SON) [62] suggest that TRPV1 has a direct influence on food intake and osmoregulation, respectively. TRPA1 has been studied less extensively than TRPV1, and recent localization studies demonstrate that TRPA1 is expressed in greater abundance in the posterior hypothalamus (PH) than the AH [63]. Comparable data on TRPV1 and A1 localization in avian brains are lacking, and we recommend exercising caution when extrapolating localization data in mammals to birds, as these receptors are both responsive to heat in birds, which is not the case in mammals.

## 3. Hypothalamic Signaling during Heat Exposure

### 3.1. Preoptic Area

The POA has projections to numerous nuclei that are activated during HAT exposure in pigeons [67]. Specifically, projections from the POA extend to the nucleus of the anterior pallial commissure (now named the nucleus of the hippocampal commissure (NHpC) [68]), the PVN, the LH, and the DMN [67]. Of these projections, the ones most commonly studied are the preoptic projections to thyrotropin-releasing hormone (TRH) neurons in the PVN. Many of the projections from the POA to other hypothalamic nuclei have been explored in regards to thermogenesis (reviewed in birds by [69]), however, relatively little has been tested regarding the activity of these projections during HAT exposure.

Most research regarding the POA and heat stress has been accomplished using rodent models. During HAT exposure, GABA-ergic neurons project from the POA to the DMN to inhibit shivering- (ST) and non-shivering (NST) thermogenesis in rats [70]. Meanwhile, glutamatergic neurons in the POA stimulate cutaneous vasodilation in mice [71]. Neurons in the POA synapse on brain-derived neurotropic factor (BDNF)-releasing neurons in the PVN [72], which may then release BDNF onto CRF-releasing neurons in rats [73,74], mice [74], and chickens [75] and TRH-releasing neurons in rats [76] and thus stimulate the release of CRF and TRH, respectively. In the blue tit (*Cyanistes coeruleus*), a songbird, the POA houses arginine vasotocin (AVT) (avian equivalent of mammalian vasopressin [77])-releasing neurons, which terminate in the NHpC [78].

In mammals, sleep is described as a thermoregulatory mechanism (For more, see [42]). In mammals, prostaglandin D2 (PGD2) is known to be the most potent sleep-inducing factor, and does so by binding to receptors within regions of the POA [79]. Some temperature-sensitive neurons in the POA respond to HAT by releasing PGD2, which in turn induces a hypothermic response [80]. Unfortunately, the thermoTRP responsible for initiating the release of PGD2 from POA neurons has yet to be identified. That PGD2 is associated with sleep and thermoregulation is fascinating. This suggests that the POA responds to HAT by inducing sleep to reduce metabolic heat production and may stimulate pathways associated with heat loss in mammals. PGD2 is also associated with an increase in neuropeptide Y (NPY) secretion in mice [81], a neuropeptide which is known to induce hypothermia in birds [82,83,84]. However, the role of PGD2 in avian sleep and thermoregulation has yet to be assessed. Further studies regarding avian exposure to HAT should explore the function of PGD2 in the avian heat response.

### 3.2. Nucleus of the Hippocampal Commissure

The NHpC is considered to be the region responsible for initiating the stress response in birds [75,85,86]. This region was activated gradually following a prolonged food deprivation stressor [75,85,86]. When exposed to HAT without fasting, the timeline drastically moved forward suggesting that the NHpC may play a role in stress signaling during heat stress, independent of nutritional state [87]. Within the NHpC, glial cells that release BDNF are bound by AVT from an unknown source [75], and it is thus possible that the AVT neurons projecting to the NHpC from the POA are responsible for this AVT. Following AVT binding, the glial cells release BDNF onto CRF neurons in the NHpC, stimulating both rapid release of CRF and rapid transcription of the gene encoding CRF [85]. Aside from binding to CRF-releasing neurons, it is hypothesized that BDNF increases in the hypothalamus in order to facilitate adaptation to an adverse environment [88]. Through adaptation, BDNF may improve survivability of those neurons and prepare them for future exposure to HAT [89]. This increase was detectable during 2 h of food deprivation, after which it decreased to control levels by 8 h [86]. In birds exposed to HAT, 40 °C, for 1 h, there was a significant decrease in CRF mRNA expression in the NHpC [87]. It is thought that NHpC CRF mRNA decreases via a negative feedback mechanism in which CRF does not activate one of its receptors, CRF receptor 1 (CRFR1), due to downregulation of CRFR1 on CRF neurons in the NHpC [85]. Although the mechanisms are not well known in aves or mammals, the POA does have an effect on food intake and homeostasis, and thus it is possible that AVT from the POA affects the NHpC during a prolonged fast [90]. However, these AVT innervations may originate from other regions such as the lateral bed nucleus of the stria terminalis (BSTL) [91]. While the direct trajectory of these NHpC CRF neurons is unclear, it is hypothesized that some of this CRF is deposited in the anterior pituitary and stimulates POMC transcription [86]. Concurrent with the downregulation of CRF in the NHpC, CRF mRNA in the PVN increases [86,87].

### 3.3. Paraventricular Nucleus

In birds, the PVN is a modulator of the stress response via CRF and AVT. Following CRF release from the NHpC, there is a slow and gradual increase in CRF produced by the PVN that persists for roughly 10 h during food deprivation in chickens [86]. CRF-ergic neurons from the PVN project to the hypophyseal portal system where CRF travels to the anterior pituitary to maintain or modulate adrenocorticotropin (ACTH) release, which in turn influences glucocorticoid release from the adrenal gland. This physiological connection is termed the hypothalamo-pituitary-adrenal axis (HPA, described in more detail below). Chicks exposed to HAT displayed an increase in hypothalamic CRF mRNA following 1 h of HAT exposure [87]. CRF mRNA abundance following heat exposure has been a controversial topic, as some birds exposed to HAT displayed no changes in CRF expression [92]. Concurrent with peak levels of CRF mRNA in food-deprived birds, AVT expression begins to rise [86]. Interestingly, while HAT exposure seems to shift the timeline of the stress response forward drastically, 1 h of heat exposure was not sufficient for increasing AVT mRNA in broiler chicks [87]. This has at least two possible explanations: (1) A single hour of heat exposure is not sufficient, as CRF was still modifying the stress response, or (2) the timeline for AVT expression in the stress cascade is independent of CRF. AVT is also released from the PVN onto the pituitary [93] where it has additional, long-lasting modulatory effects on corticotrophs [94]. Alterations in AVT expression are associated with changes in blood composition and shifts in osmotic pressure [95]. That food deprivation without withholding water stimulated AVT release, and an hour of HAT exposure did not, suggests that there may be an alternative mechanism through which AVT is stimulated that is independent of osmotic pressure or dehydration. This hypothesis is further supported by evidence in chickens that HAT increased plasma AVT independent of osmotic pressure [96]. It is possible that AVT plays a role in reducing body temperature in response to HAT, as elevated plasma AVT elicited a hypothermic response in pigeons [97].

As described later in this review, in addition to ACTH and the stress response, several other hormonal cascades associated with the anterior pituitary are involved in thermoregulation, including thyroid hormones (via thyroid-releasing hormone) and prolactin, whose production and release are also regulated by hypothalamic mediators.

### 3.4. Arcuate Nucleus

The ARC projects to several nuclei in the hypothalamus to mediate food intake, including the PVN and LH [98]. Studies involving different genetic stocks of chickens have brought about controversy over NPY, an ARC-derived neuropeptide, and its role in the response to HAT. Following heat exposure, chickens have increased NPY mRNA [99,100], decreased NPY mRNA [87], or no change when compared to controls [92]. Similarly, broiler chicks injected with NPY via intracerebroventricular (ICV) injection during heat exposure had a diminished orexigenic response to the NPY [87], while layer-type chicks ICV injected with NPY during heat exposure responded similarly to thermoneutral chicks [82]. NPY treatment reduced body temperature in layer-type chickens [82,83,84], however this has yet to be explored in broilers. These data collectively suggest that the hypothermic effect of NPY may not be present in broilers. The reduction in NPY abundance and function during heat stress is a plausible explanation for why food intake is reduced. In mice, CRF-releasing neurons from the PVN project to the median eminence through the ARC [101]. Some of these CRFergic neurons in mice have axon collaterals that synapse onto NPY neurons, releasing CRF which binds to the CRFR1 receptor causing inhibition of these neurons [102]. It is possible that this also occurs in birds.

## 4. Extrahypothalamic Endocrine Consequences

### 4.1. Corticotrophs and Corticosterone

The remainder of this review will focus on subpopulations of endocrine cells in the anterior pituitary and their associated hormonal cascades, including corticotrophs, thyrotrophs, and lactotrophs. Starting with the stress cascade, CRF, and corticotrophs, once CRF and AVT have arrived in the anterior pituitary, they bind to receptors on corticotrophs to stimulate the release of ACTH into the blood stream [93,103]. CRF, and to a lesser degree, AVT, are both capable of stimulating ACTH release in birds [104], but together have an additive effect through dimerization of the CRFR1 and AVT receptor 2 (VT2R) [105]. ACTH then circulates and stimulates the release of corticosterone (CORT) from the avian adrenal cortex. The avian adrenal gland differs in structure and organization from mammals [106,107], however, to our knowledge, the implications of these differences in the stress response are unknown. Exposure to HAT caused a drastic increase in plasma CORT in chickens [108,109], turkeys [110], and pigeons [111]. This is interesting, as injection of glucocorticoids is known to stimulate food intake in birds including doves [112], sparrows [113], quail [114], and chickens [115,116]. Despite an increase in food intake, glucocorticoid-injected chickens [115,116] weighed less, and because of a lack of supporting studies it is unclear whether weight loss was steroid-induced. CORT stimulates NPY gene expression in chickens [117], which may contribute to its orexigenic effects. Based on the effect of CRF on NPY neurons in the ARC, during HAT exposure, the inhibitory effect of CRF signaling likely overpowers the stimulatory effect of CORT, or may diminish the number of glucocorticoid receptors on NPY neurons.

Plasma CORT concentrations remain elevated in chickens exposed to chronic heat stress for up to 7 days of heat stress, and return to control levels by day 14 of heat stress [118]. The two weeks of heat stress were associated with a reduction in hypothalamic NPY mRNA and protein expression [118]. Visualization of the hypothalamus of heat stressed-birds revealed disintegration of blood vessels and neurons [118], suggesting that long-term physiological impacts of chronic heat stress may be the consequence of heat-induced brain tissue damage. Prolonged CORT expression and lasting reductions in NPY likely contribute to reductions in body weight and food intake in broilers exposed to chronic heat stress [12]. That the hypothalamus also plays a major role in integrating signals to regulate metabolism suggests that heat-induced damage to the hypothalamus may partially explain the persistent metabolic dysfunction [12]. Research on the effects of chronic heat stress in the avian brain is quite limited [119] in spite of the practical importance of such knowledge.

CORT signaling affects other factors involved in thermoregulation, including thyroid hormones. In broiler chickens, daily injections of CORT reduced the concentration of plasma triiodothyronine (T_3_) [120]. Additionally, T_3_ concentrations were reduced during acute thermal exposure in pigeons [121] and broilers [122], while plasma thyroxine (T_4_) concentrations were increased in broilers [122]. In contrast, injections of CORT did not affect plasma T_3_ in rodents [123,124], and when mice were exposed to chronic stressors that were categorized as mild, plasma CORT increased within 1 week, while T_3_ was unaffected until week 4 of exposure [125]. That T_3_ concentrations are differentially affected by CORT in rodents and birds suggests divergence in these systems during evolution. This may be associated with alterations in metabolic needs in birds, as birds rely on thyroid hormones to maintain fat deposition prior to migration [126], after which responsibility for energy deposition control is assumed by CORT just prior to, and during flight [127,128].

The shift from thyroid hormones to CORT near the time of migration suggests that the effect of HAT exposure may be affected by the time of year, as this determines which hormone is mediating energy deposition and expenditure. Such changes may not be of as much practical importance in a commercial poultry setting but could be extremely important to consider in the wild as a response to climate change.

### 4.2. Thyrotrophs and Thyroid Hormones

The thyroid hormones T_3_ and T_4_ play a critical role in thermoregulation [129,130,131] and metabolism [132,133,134,135] in birds and mammals. However their role in regulating food intake in birds is unclear. In rodents, T_3_ affects food intake, and a receptor for T_3_, thyroid receptor β (TRβ), exists in the ventromedial hypothalamic nucleus (VMN) [136]. Injection of T_3_ into the VMN increased food intake in rats [137]. Knockout of TRβ also elicits hyperphagia via an increase in NPY expression in the ARC [136]. It is hypothesized that TRβ is responsible for mediating food intake independent of changes in metabolic function, as pair-fed mice remained lean [136]. Similarly, T_3_ is responsible for mediating the action of uncoupling protein 2 (UCP2) in NPYergic neuronal mitochondria in the ARC [138]. Increased T_3_ in the ARC stimulates UCP2 activity, increasing excitability of NPY neurons and thus the release of NPY [138]. Interestingly, microinjections of T_3_ into the ARC are not sufficient to stimulate food intake [137], suggesting that T_3_ may need to act upon multiple hypothalamic nuclei in order to be orexigenic. Although direct appetite-regulatory roles are unknown, heat stress is associated with changes in thyroid hormone concentrations in birds. Broiler chickens and turkeys exposed to HAT had lower plasma concentrations of T_3_ and increased plasma T_4_ [139,140,141,142,143]. It is tempting to speculate that the decreased T_3_ is associated with the reduction in food intake and metabolic heat production. Reduced T_3_ may be related to the reduction in NPY mRNA observed in chickens [87] via reduced UCP activity. Birds lack the respective orthologs of the three uncoupling proteins (UCP1, 2 and 3) found in mammals, and instead have a single UCP called avian UCP (avUCP) that shares 70% sequence similarity with UCP2 and 3 [144]. AvUCP is almost exclusively expressed in the skeletal muscle [144], with trace amounts in the heart, liver, lung, and kidney [145]. Interestingly, avUCP was not detected in the brain [145], but it is possible that avUCP is expressed in specific regions, such as various hypothalamic nuclei, and overall tissue volume diluted out distinct areas of expression. This is further supported by the report that avUCP is expressed ubiquitously like UCP2 [144], which is expressed in the hypothalamus of mammals. The role of thyroid hormones, the TRβ receptor, and avUCP should be explored in the avian hypothalamus, especially in the VMN and ARC.

Exposing broiler chicks to a heat stressor at an early age, within the first week of life, reduced the mortality rate when those birds were exposed again 40 days later [10]. Chicks exposed at day 5 did not lose weight and were more feed efficient [10]. That thyroid hormones modulate metabolic function and thermoregulation suggests that acclimatization at a young age might prepare the bird for future heat stressors through this system. However, chicks exposed to a heat stressor for the first 3 days post-hatch (acclimatization) or on day 4 alone (stressor) had similar plasma T_3_ and T_4_ concentrations as controls [122]. Additionally, chicks that underwent radiothyroidectomy on day of hatch and were then exposed to a heat stressor at 20 days post-hatch survived longer than controls, but did not differ in body weight [146]. Collectively, these results are perplexing, as they suggest that (1) thyroid hormones are not involved in the adaptation to heat stressors in birds and (2) that alterations in thyroid hormones are not responsible for the observed changes in body weight.

For future studies involving T_3_ and food intake in birds, it should be considered that T_3_ may have an age-dependent effect in birds. During the first 2 weeks of life post-hatch, chicks injected with T_3_ are immune to the thermogenic effects of T_3_ [147]. It is hypothesized that this is because at hatch T_3_ concentrations are exceptionally high, and this is likely to increase metabolism in order to provide energy for hatching [148]. Additionally, while exogenous T_3_ does elicit a thermogenic response in older birds, the range of doses that are effective is questionable. Contradicting data suggest that most doses used in the literature may not be sufficient as they do not surpass biological concentrations (reviewed by [149]). Lastly, although not tested in birds, it should be noted that injections of T_3_ into the ARC do not affect food intake [137], and ICV injections of T_3_ do not influence neuronal activity in the ARC [150] of rodents. However, intraperitoneal (IP) injections of T_3_ do stimulate the ARC of rodents [138]. To our knowledge, the reasoning behind the difference in response to ICV vs. IP injections is unknown in any species. We therefore emphasize exercising caution when extrapolating the effects of thyroid hormones to birds, as doses significantly below and above the physiological concentrations can lead to misleading interpretations of their role in physiology, and this may also vary across the lifespan of the bird.

### 4.3. Lactotrophs and Prolactin

In addition to CORT, prolactin (PRL), a hormone produced by lactotrophs in the pituitary, may play a role in fat deposition in migratory birds, depending on the time of day [151], however this effect is not present in broiler-type chickens [152]. Moreover, a multitude of studies describe heat stress-induced elevations in plasma prolactin in various ruminant species such as cattle and sheep and a lack of effective thermoregulation in the absence of prolactin. During exposure to heat stress, plasma PRL concentrations increased in ewes [153] and cattle [154,155,156]. Suppression of PRL release impaired thermoregulation in ewes exposed to acute [157] and chronic [158] heat stressors. In both studies, suppression of PRL release was associated with increased rectal temperatures and increased respiration rates. Interestingly, during heat stress, the sensitivity of PRL release to TRH was unaffected in sheep, and reductions in food intake did not alter PRL secretion during heat stress in cattle [154]. This suggests that changes in PRL concentrations during heat stress occur independent of nutritional state and alterations in metabolism. It is hypothesized that changes in PRL concentration during heat stress are mediated at the level of the hypothalamus [153].

In heat-stressed poultry, prolactin might be associated with heat stress-induced changes in egg laying and incubation behavior. In turkeys exposed to heat stress, egg laying was terminated, and plasma PRL concentrations and incubation behaviors increased [159]. Exposure to heat stress also caused an increase in plasma PRL concentrations in laying hens [160] and PRL gene expression in the hypothalamus of broilers [99]. Chemically induced hypothyroidism increased plasma PRL concentrations similar to heat stress-exposed laying hens, and administration of T_4_ attenuated this effect with PRL concentrations near basal levels [160]. To our knowledge, the role of PRL in avian thermoregulation has yet to be explored, but these data suggest that in the absence of thyroid hormones, PRL compensates and in turn mediates thermoregulation. However, the secretagogue of PRL, prolactin-releasing peptide (PrRP), reduced rectal temperature in laying hens [84] and it is thus plausible that this effect is mediated in part by subsequent PRL secretion. This hypothesis supports that heat stress-induced alterations in PRL are mediated at the hypothalamus. How PRL mediates thermoregulation or metabolism is not well understood in any species. Thus, we emphasize the need to explore PRL biology during heat stress in birds and mammals (for more, see [161]).

## 5. Summary

Heat stress and heat exposure are challenges in the poultry industry that are known to be the leading causes of production losses. Unfortunately, the exploration of thermoregulatory mechanisms in birds is limited, and the majority of research regarding thermoregulation in mammals focuses on the pathways mediating thermogenesis via the POA. The thermoTRPs TRPV1 and TRPA1 respond to heat stimuli in birds, yet both have been studied in the context of development of bird repellants via noxious stimuli. That these ion channels respond in a different manner than in mammals suggests differences in their signaling pathways. Lastly, localization of these ion channels has yet to be explored in the avian brain, and little is known about the avian POA regarding thermoregulation during HAT. It is suggested that these ion channels and the POA and its neuronal projections and signaling be explored in birds, especially the pathways involving appetite and stress responses to heat exposure.

Elucidation of a role for the NHpC in mediating heat stress response pathways underscores the strong possibility that the avian stress response originates in a brain region different from mammals. Unfortunately, due to its novelty, little is known regarding how the signals manifest or if the NHpC is involved in thermoregulation. It is hypothesized that the POA projects and releases AVT onto the NHpC, suggesting that the POA may signal that the ambient temperature is dangerous and thus stressful, but future studies should aim to identify this mechanism. Furthermore, studies should explore the role of CORT in the NHpC, and its roles in mediating NPY and CRF expression during heat stress, as these all seem to be involved in the heat stress response, as well as heat-induced anorexia.

Thyroid hormones are associated with thermogenesis in birds, and it is well documented that heat exposure reduces T_3_ abundance in plasma. In rodents, thyroid hormones likely play a role in stimulating food intake independent of metabolic activity. However, the role of thyroid hormones in mediating food intake in birds has yet to be explored. In poultry, thyroid hormones are differentially abundant in the plasma of young chicks and older birds. This is likely due to metabolic needs of hatching, and due to a greater abundance of cold-sensitive neurons in younger chicks which may drive T_3_ in order to elicit a thermogenic response. That chicks exposed to HAT within the first week of life appear to be more stress resilient when exposed later in life may be related to this mechanism, and thus exploration of epigenetic modifications in genes associated with T_3_ production, as well as changes in thermo-sensing neurons in the avian brain, should be explored. Lastly, prolactin, which is also part of a cascade that involves hypothalamic regulation and hormonal release from the anterior pituitary, may also play an important role in thermoregulation and energy metabolism in birds, but requires further study, especially in the context of heat stress and the relationship to other hormonal pathways.

## Data Availability

In the present review there were no data analyzed. All data and reports mentioned were from cited articles found via search engines such as PubMed, Elsevier, and Google scholar.
